# Characterization of the complete chloroplast genome of *Handroanthus chrysanthus* (Bignoniaceae)

**DOI:** 10.1080/23802359.2022.2102445

**Published:** 2022-08-17

**Authors:** Hong-Ze Liao, Man-Man Sun, Hao Zhou, Xiu Liu

**Affiliations:** a Key Laboratory of Protection and Utilization of Marine Resources, Guangxi Minzu University, Nanning, China; b Guangxi Key Laboratory of Special Non-wood Forest Cultivation and Utilization, Guangxi Forestry Research Institute, Nanning, China

**Keywords:** *Handroanthus chrysanthus*, Bignoniaceae, chloroplast genome, phylogenetic analysis

## Abstract

*Handroanthus chrysanthus* is a deciduous broadleaved species with ecological and medicinal value. Here, the complete chloroplast genome of *H. chrysanthus* is characterized to investigate its phylogenetic position in Bignoniaceae. The chloroplast genome is 159,437 bp in size with GC content of 38.1%, including a large single copy region of 85,659 bp, a small single copy region of 12,824 bp and a pair of inverted repeats of 30,477 bp. It encodes 132 genes, including 87 protein-coding genes, 37 tRNA genes, and 8 rRNA genes. Based on current available chloroplast genome sequences, the phylogenetic analysis indicated that *H. chrysanthus* is closely related to *Tabebuia nodosa*.

*Handroanthus chrysanthus* (Jacq.) S. O. Grose (synonym *Tabebuia chrysantha* (Jacq.) G. Nicholson, 1877) is the species of the genus *Handroanthus* within the family Bignoniaceae (Grose and Olmstead 2007). As a deciduous broadleaved tree with brilliant yellow flowers, it is widely used in gardening in tropical and subtropical areas, and its stem extract possesses antimicrobial, anti-inflammatory and anticancer properties (Panda et al. 2019). In this study, we described and characterized the complete chloroplast genome of *H. chrysanthus* to better understand its genomic structure and phylogenetic relationship in Bignoniaceae.

The fresh leaves of *H. chrysanthus* were collected from Guangxi Forestry Research Institute, Nanning, China (N22.92°, E108.35°), and the specimens were conserved in Guangxi Minzu University (Hao Zhou, zhou.hao.gxun@foxmail.com) under the voucher number LCY20190606. The total genomic DNA was extracted following a modified CTAB protocol (Allen et al. 2006) and sequenced using NovaSeq 6000 system (Illumina, San Diego, CA, USA). In total, about 4.4 Gb of raw reads with 14,491,668 clean paired-end reads were generated. The chloroplast genome was assembled using SPAdes v3.13.1 (http://cab.spbu.ru/software/spades/) (Bankevich et al. 2012) and annotated with PGA software (https://github.com/quxiaojian/PGA) (Qu et al. 2019), then deposited into GenBank under the accession number ON243876.

The chloroplast genome of *H. chrysanthus* is 159,437 bp in length, with an overall GC content of 38.1%. It comprises a large single copy region of 85,659 bp and a small single copy region of 12,824 bp, which were separated by a pair of inverted repeats of 30,477 bp. A total of 132 genes were predicted, including 87 protein-coding genes, 37 tRNA genes, and 8 rRNA genes.

There was only one complete chloroplast genome of *Handroanthus* species (*Handroanthus impetiginosus*) well-characterized before (Sobreiro et al. 2020), and it might share a close relationship with *H. chrysanthus*. Unfortunately, the chloroplast genome sequence of *H. impetiginosus* was unavailable in public database. To reveal the phylogenetic position of *H. chrysanthus*, the complete chloroplast genomes of 16 other species from Bignoniaceae and one species (*Aloysia citrodora*) from Verbenaceae served as an outgroup were introduced for phylogenetic analysis. The sequences were aligned by MAFFT v7.490 (https://mafft.cbrc.jp/alignment/software/) (Katoh and Standley 2016) and a maximum-likelihood tree was constructed by IQ-TREE 2 (http://www.iqtree.org) using the TVM + F + I + G4 model with 1000 bootstrap replications (Minh et al. 2020). Based on current available chloroplast genome sequences, the phylogenetic analysis showed that *H. chrysanthus* was closest to *Tabebuia nodosa* (Figure 1).

**Figure 1.**
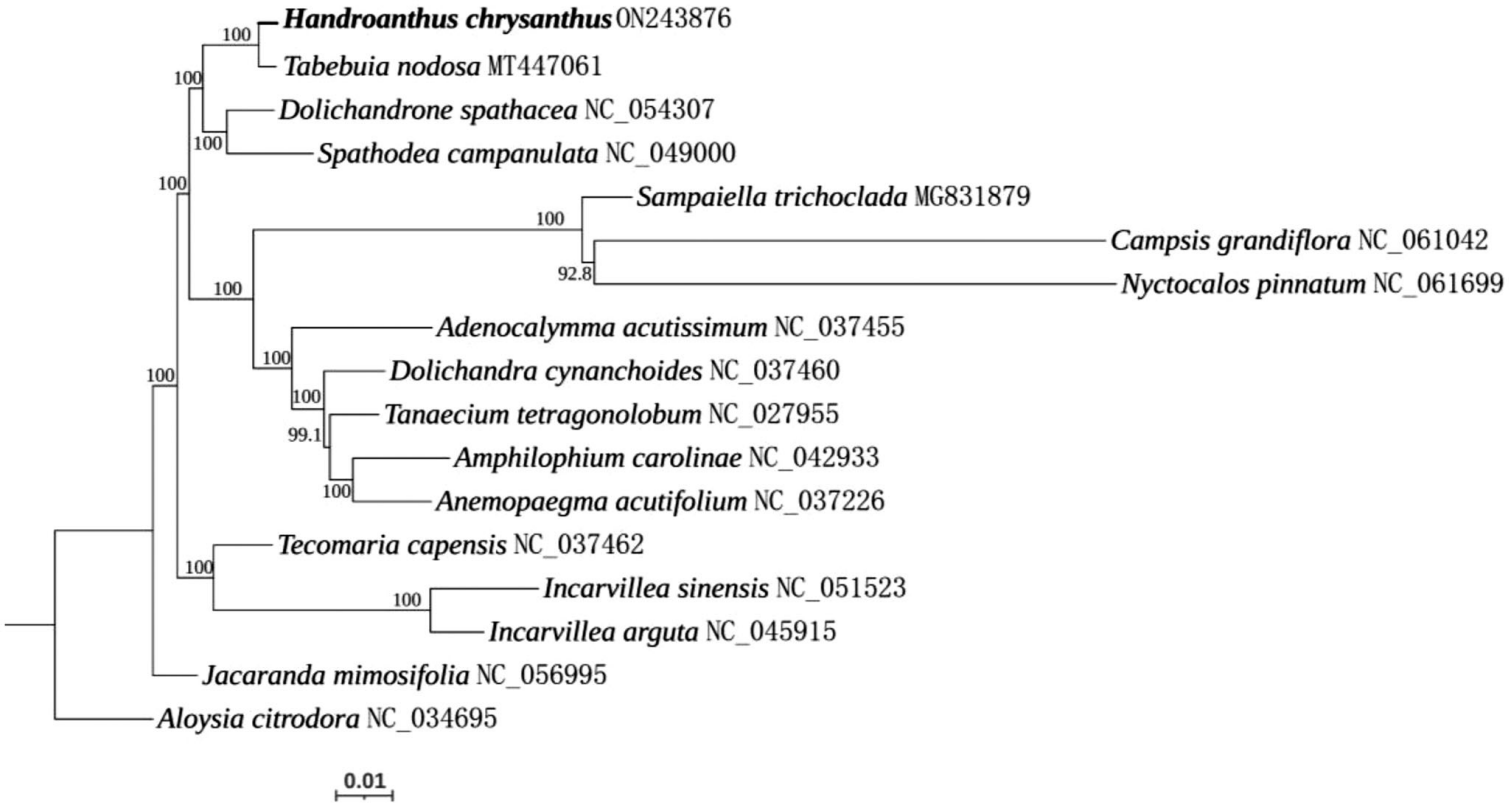
The maximum-likelihood phylogenetic tree based on the 16 chloroplast genomes of Bignoniaceae. *Aloysia citrodora* (Verbenaceae) served as an outgroup.

## Author contributions

X. L. and H. Z. L. conceived and designed the experiments; H. Z. L., M. M. S., and H. Z. performed the experiments and analyzed the data; H.Z.L. and X. L. wrote the paper. All authors have read and approved the final manuscript to be published, and agree to be accountable for all aspects of the work.

## Data Availability

The data support the findings of this study are openly available in NCBI GenBank (https://www.ncbi.nlm.nih.gov) under the accession number ON243876. The associated BioProject, SRA, and Bio-Sample numbers of the raw sequencing data of *Handroanthus chrysanthus* are PRJNA826917, SRR18765829, and SAMN27591411, respectively.
